# Dentinogenic Effect of BMP-7 on Wharton’s Jelly Mesenchymal Stem Cells Cultured in Decellularized Dental Pulp

**DOI:** 10.3390/ijms262311760

**Published:** 2025-12-04

**Authors:** Nur Athirah Ahmad Shuhaimi, Dalia Abdullah, Farinawati Yazid, Sook Luan Ng, Nurul Inaas Mahamad Apandi, Nur Azurah Abdul Ghani

**Affiliations:** 1 Department of Restorative Dentistry, Faculty of Dentistry, Universiti Kebangsaan Malaysia, Kuala Lumpur 50300, Malaysia; p131245@siswa.ukm.edu.my (N.A.A.S.); daliaabdullah@ukm.edu.my (D.A.); 2 Department of Family Oral Health, Faculty of Dentistry, Universiti Kebangsaan Malaysia, Kuala Lumpur 50300, Malaysia; 3 Department of Craniofacial Diagnostics and Biosciences, Faculty of Dentistry, Universiti Kebangsaan Malaysia, Kuala Lumpur 50300, Malaysia; nurulinaas@ukm.edu.my; 4 Department of Obstetrics and Gynecology, Faculty of Medicine, Universiti Kebangsaan Malaysia, Jalan Yaakob Latif, Bandar Tun Razak, Kuala Lumpur 56000, Malaysia

**Keywords:** cell proliferation, dentin sialophosphoprotein, dentin matrix protein-1, odontogenic marker, regenerative endodontic therapy

## Abstract

Decellularized tissue scaffolds mimic the native pulp-dentin microenvironment and support the odontogenic development of stem cells. This study investigated the dentinogenic effect of Wharton’s Jelly Mesenchymal Stem Cells (WJMSCs) in decellularized human dental pulp (DHDP) with bone morphogenic protein-7 (BMP-7) at three concentrations: 0 ng/mL (control), 25 ng/mL, and 50 ng/mL. The effects of BMP-7 were evaluated by histological examination, WJMSC viability using AlamarBlue, dentinogenic gene expression by qPCR, and dentinogenic protein expression by ELISA. By day 21, all three groups exhibited cell distribution along the pore surfaces of DHDP, followed by the presence of a collagen matrix in the tissue. WJMSC viability treated with 25 ng/mL and 50 ng/mL showed a statistically significant increase on days 7, 14, and 21 compared to the control group (*p* < 0.05). Gene expression analysis of dentin sialophosphoprotein (DSPP) and dentin matrix protein-1 (DMP-1), and odontogenic marker (Runx2) revealed 25 ng/mL BMP-7 resulted in significantly higher expression levels for DMP-1 and Runx2 on day 21 compared to control and 50 ng/mL BMP-7 group (*p* < 0.05). DSPP and DMP-1 protein expressions also showed trends similar to those of gene expressions. BMP-7 (25 ng/mL) can maintain cell viability and promote dentinogenic effects of WJMSC in the DHDP scaffold.

## 1. Introduction

The dental pulp plays an important role in maintaining the vitality and functionality of teeth. The pulp and the surrounding dentine form the dentin-pulp complex, which is essential for sensory perception and nutrient supply [1]. The dental pulp is vulnerable to damage from various insults such as caries and trauma, which can lead to pulp necrosis and, ultimately, tooth loss if left untreated. Root canal treatment (RCT) involves the removal of necrotic pulp tissue and its replacement with synthetic materials, which fail to restore the biological functions of the pulp. This limitation is particularly critical in young permanent teeth, where pulp loss can disrupt root development and compromise long-term dental health [2]. Failure rates associated with RCTs range from 5% to 18%, depending on diagnostic guidelines and follow-up time [3,4,5,6]. Different approaches, therefore, need to be explored to address the limitations of RCT by restoring the natural pulp-dentin structure rather than simply filling it with non-living materials.

Regeneration is defined as a process of replacing damaged tissues with new tissues of similar biological properties and organization, enabling restoration of their full functions [7,8]. Regenerative endodontic therapy (RET) aims to regenerate pulp tissue, thereby preserving the tooth’s vitality. The recommended clinical procedure using the cell-homing technique was outlined by the American Association of Endodontology [9]. The aim is to recruit and promote the host cells (stem cells from the apical papilla at the root end) to migrate into the canal and regenerate. Favourable clinical outcomes, such as continuous root maturation and healing of periapical pathology, were observed in immature teeth [10]. However, histological analysis revealed that the regenerated tissue following RET was mainly mineralized [11], suggesting repair or replacement rather than true regeneration. Failed cases showed tissues that were less organized and distinct from pulp tissues [10]. The failure could be attributed to an insufficient number of stem cells recruited to the pulp space [12]. In some instances, cell-homing RET resulted in ectopic periodontal and periapical tissues in the canal space rather than pulp tissue, possibly due to the migration of neighbouring periodontal mesenchymal stem cells from the periapical tissues to compete for tissue regeneration [12].

While RETs represent a significant step forward in treating immature necrotic teeth to promote regeneration of the pulp-dentin complex, clinical results indicate a steady failure rate that warrants attention. Recent high-quality studies suggest that, despite overall favourable success rates of 88.7% to 91.5%, RETs have failure rates ranging from 8.5% to 11.3% across various study designs and groups. The most comprehensive evidence comes from a meta-analysis by Goyal et al. (2017), which examined 412 immature teeth and reported an 8.5% failure rate [13]. Similarly, Chrepa et al. (2020) found an 11.3% failure rate in their detailed 24-month study involving 62 teeth [14]. Kim et al. (2021) reported an 8.9% failure rate in their retrospective review of 45 cases [15]. Most recently, Li et al. (2023) conducted a systematic review of 512 teeth from 18 studies, calculating a pooled failure rate of 10.4% [16]. These consistent findings across different research methods, including single-centre cohorts and meta-analyses, confirm that current REP protocols have a failure rate of 9% to 11% [13,16]. The failure often manifests as persistent periapical lesions requiring conventional retreatment. This evidence is important for weighing the benefits of REPs against their drawbacks when considering treatments for immature permanent teeth.

There is a need to find a predictable clinical protocol. The tissue engineering triad provides the framework for identifying appropriate cells, scaffolds, or growth factors to regenerate functional pulp tissue [1]. Among various stem cell sources, mesenchymal stem cells (MSCs) derived from Wharton’s Jelly have attracted significant attention for their high proliferative capacity, multipotency, and low immunogenicity [17]. Wharton Jelly’s mesenchymal stem cells (WJMSCs) can differentiate into various cell types, including odontoblasts, which are essential for dentin formation, making them ideal for regenerative endodontics [18]. Furthermore, the abundant supply of umbilical cord tissue and the ease of isolation make WJMSCs a practical alternative to dental pulp stem cells (DPSCs), which are limited in availability [19].

The ethical implications of stem cell sourcing are a critical consideration in regenerative dentistry. WJMSCs have become an ethically non-controversial alternative to embryonic stem cells [20]. Unlike embryonic sources, which raise significant moral debates about the destruction of human embryos, WJMSCs come from the umbilical cord. This tissue is typically discarded as medical waste after birth, and its procurement requires only informed maternal consent [21,22]. As Charitos et al. (2021) [20] point out, perinatal tissues, such as Wharton’s Jelly, avoid the major ethical dilemmas associated with embryonic stem cells while still offering similar regenerative potential. This ethical advantage is highlighted by their immunological privilege and non-tumorigenic properties, making them especially suitable for autologous and allogeneic dental applications [20]. Importantly, as Castaldi et al. (2019) stress, collecting WJMSCs poses no additional risk to donors and raises no legal issues, as the umbilical cord would be discarded after birth [21]. Recent literature, including Sharma and Maurya’s, (2024) [22] review, shows a growing consensus that positions WJMSCs as an ethically sound and clinically promising source for dental tissue engineering. They combine embryonic-like plasticity with the practical and moral advantages of adult stem cell sources [22].

Scaffolds, another critical component of tissue engineering, provide a three-dimensional (3D) microenvironment that supports cell attachment, proliferation, and differentiation. Decellularized human dental pulp (DHDP) has been proposed as an ideal scaffold due to its ability to retain the native extracellular matrix (ECM) architecture, which is crucial for guiding tissue regeneration [23]. The decellularization process removes cellular components while preserving the ECM, thereby reducing immunogenicity and providing a conducive environment for cell growth and differentiation [24].

Growth factors, such as bone morphogenetic protein-7 (BMP-7), regulate stem cell behaviour and can promote tissue regeneration. BMP-7 has been shown to induce osteogenic and dentinogenic differentiation in MSCs, making it a promising candidate for pulp regeneration [25]. Previous studies have demonstrated the potential of BMP-7 in promoting dentin formation in DPSCs, but its application in WJMSCs for pulp regeneration has not been thoroughly investigated [26]. This study explored the effect of BMP-7 in promoting dentinogenesis in WJMSCs cultured in DHDP scaffolds. The findings from this research would provide supporting data on the use of WJMSCs with DHDP as a scaffold, treated with BMP-7, for the pulp regeneration procedure.

## 2. Results

### 2.1. MTT Assays

**Figure 1:**
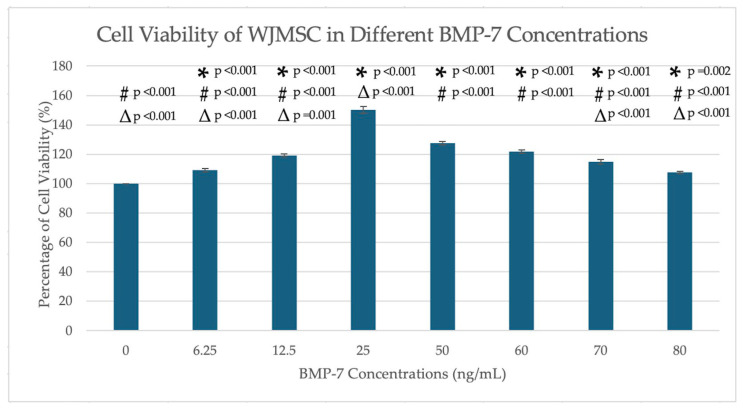
Cell viability (%) of WJMSCs after treatment with different concentrations of BMP-7. Cell viability was normalized to the control group (0 ng/mL BMP-7). Data are presented as mean ± SEM. Exact *p* values from post hoc analysis are displayed on the figure. * *p* < 0.05 indicates a statistically significant difference compared with the control. # *p* < 0.001 indicates a statistically significant difference compared with 25 ng/mL BMP-7. ∆ *p* < 0.05 indicates a statistically significant difference compared with 50 ng/mL BMP-7.

MTT (3-(4,5-Dimethylthiazol-2-yl)-2,5-Diphenyltetrazolium Bromide) assays revealed that BMP-7 significantly enhanced WJMSC viability in a dose-dependent manner. Among the tested concentrations (0–80 ng/mL), 25 ng/mL BMP-7 exhibited the highest cell viability, showing a significant increase compared to all other BMP-7 concentrations and the control group (*p* < 0.001) (Figure 1). Furthermore, all BMP-7-treated groups showed significantly higher viability than the control group (*p* < 0.05).

### 2.2. Histological Evaluation of Human Dental Pulp and Decellularized Scaffold

**Figure 2:**
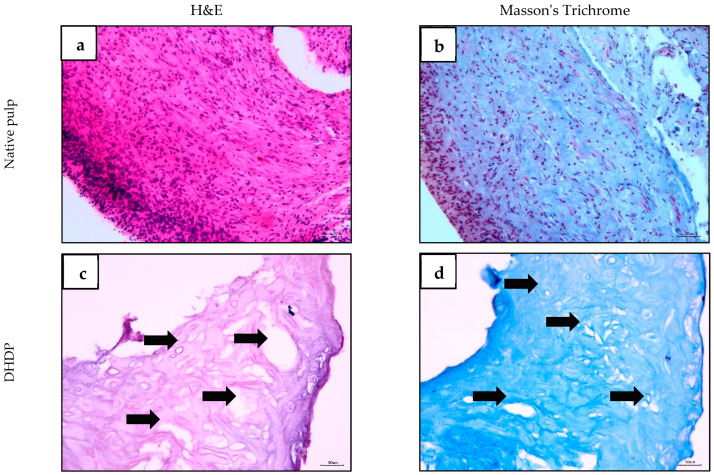

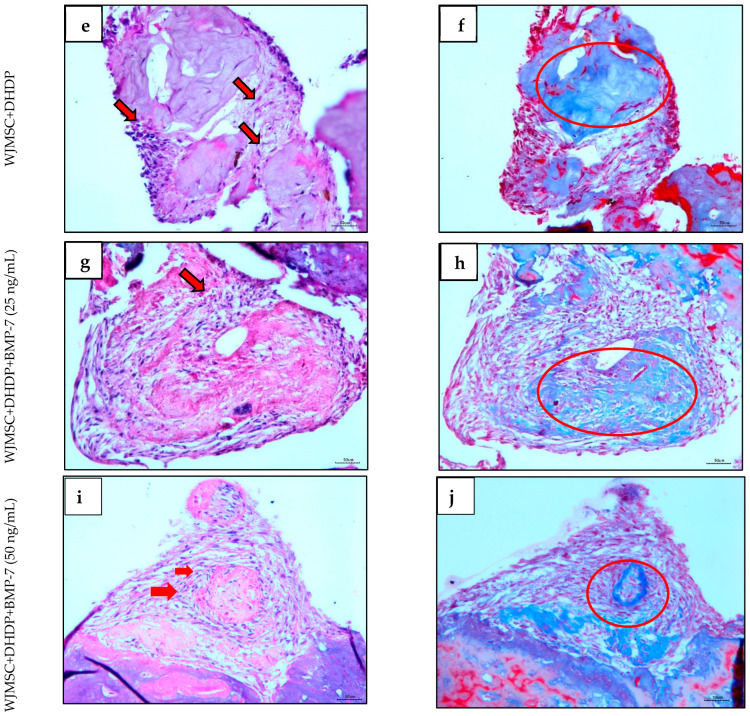
Histological observations using H&E staining (**a**,**c**,**e**,**g**,**i**) and Masson’s trichrome staining (**b**,**d**,**f**,**h**,**j**) of human pulp tissue (**a**,**b**), DHDP scaffold (**c**,**d**), DHDP scaffold repopulated with WJMSC without BMP-7 treatment (0 ng/mL) (**e**,**f**), and DHDP scaffold repopulated with WJMSC with two concentrations of BMP-7, namely 25 ng/mL (**g**,**h**) and 50 ng/mL (**i**,**j**) on day 21 with 100× magnification. (**c**,**d**) DHDP scaffold showed no cellular presence, confirming successful decellularization. The scaffold exhibited a porous structure (indicated by black arrows). H&E staining revealed the presence of fibroblasts (red arrows) in all groups (**e**,**g**,**i**). Masson*’*s Trichrome staining demonstrated the presence of collagen fibers (blue staining in red circle) in the ECM of all groups (**f**,**h**,**j**). Scale bar = 50 µm.

Sections of non-decellularized human dental pulp stained with hematoxylin and eosin (H&E) showed its typical morphology, with abundant various cell types, including fibroblasts and immune cells in mesenchymal connective tissue and blood vessels containing red blood cells (Figure 2a). Layers of odontoblasts can be recognized by the irregular short or tall columnar cells (Figure 2a). DHDP samples showed no cellular presence, confirming successful decellularization (Figure 2c,d). The samples exhibited porous structures. Masson’s Trichrome staining demonstrated the presence of collagen fibres (blue staining) in the ECM of the non-decellularized human dental pulp and the DHDP (Figure 2b,d).

### 2.3. Characterization of WJMSC

**Figure 3:**
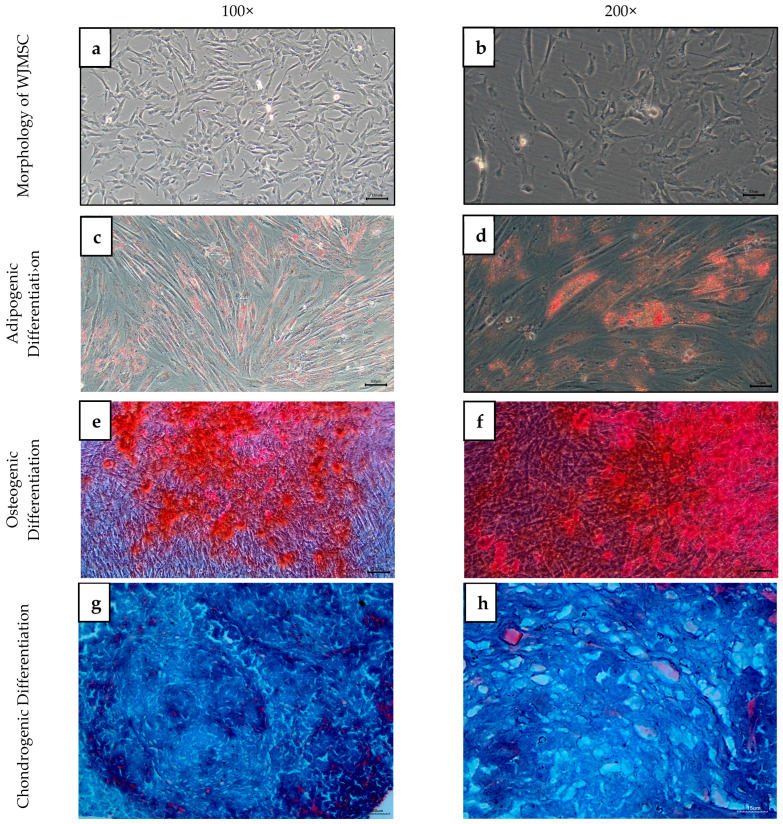
Photomicrographs of WJMSCs. Cells at passage 3 appeared spindle-shaped with fibroblast-like morphology and adhered to the plastic flask (**a**,**b**). WJMSCs exhibited intracellular lipid accumulation for adipogenic differentiation (**c**,**d**) (Stain: Oil Red O). Observation of calcium deposition for osteogenic differentiation (**e**,**f**) (Stain: Alizarin Red) and presence of sGAG in the ECM in blue, and fibroblast in red purplish for chondrogenic differentiation (**g**,**h**) (Stain: Alcian Blue and Nuclear Fast Red). Phase-contrast microscopic images (**a**,**c**,**e**) were captured at 100× magnification (scale bar = 100 µm), while images (**b**,**d**,**f**) were captured at 200× magnification (scale bar = 50 µm). Light microscopic image (**g**) was taken at 100× magnification (scale bar = 50 µm) and image (**h**) at 200× magnification (scale bar = 15 µm).

WJMSCs exhibited a spindle-shaped, fibroblast-like morphology, forming a monolayer that adhered to the plastic culture flask. The cells reached 70–80% confluence, as observed under an inverted microscope at 100× and 200× magnification (Figure 3a,b). This morphology was consistent with the typical characteristics of mesenchymal stem cells (MSCs), confirming the successful isolation and culture of WJMSCs.

The multipotency of WJMSCs was confirmed through their ability to differentiate into adipogenic, osteogenic, and chondrogenic lineages. After 14 days of induction, WJMSCs exhibited intracellular lipid accumulation for adipogenic differentiation, as evidenced by positive staining with Oil Red O (Figure 3c,d). The presence of orange-red lipid vacuoles confirmed the adipogenic potential of WJMSCs. As for osteogenic differentiation, by day 28, WJMSCs demonstrated calcium deposition, as indicated by Alizarin Red staining (Figure 3e,f). The formation of mineralized nodules confirmed the osteogenic differentiation potential of WJMSCs. After 21 days of chondrogenic induction, WJMSCs formed pellets that stained positively with Alcian Blue and Nuclear Fast Red (Figure 3g,h). The presence of sulfated glycosaminoglycans in the extracellular matrix confirmed chondrogenic differentiation in blue, while the presence of fibroblasts in red purplish colour.

**Figure 4:**
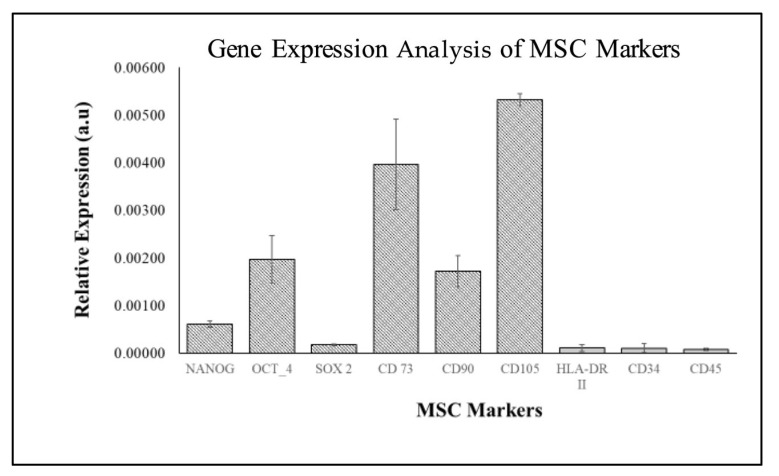
Characterization of WJMSC using MSC markers: NANOG, OCT-4, SOX2, CD73, CD90, CD105 (positive MSC markers), HLA-DR II, CD34, and CD45 (negative MSC markers). Data (*n* = 6) are expressed as mean ± SEM.

Gene expression analysis by using MSC markers revealed that WJMSCs expressed high levels of MSC-positive markers (CD73, CD90, and CD105) and low levels of MSC-negative markers (HLA-DRII, CD34, and CD45) (Figure 4). These findings confirm the mesenchymal stem cell characteristics of WJMSCs. However, compared with OCT-4, the expression levels of NANOG and SOX2 were low in WJMSC.

### 2.4. Histological Analysis of Repopulated DHDP with and Without BMP-7

The H&E staining of all samples in the control and experimental groups (0 ng/mL, 25 ng/mL, and 50 ng/mL) at day 21 revealed healthy fibroblasts, identifiable by their spindle-shaped morphology (Figure 2e,g,i). The cells were well dispersed and integrated into the scaffold’s ECM.

Masson’s Trichrome staining demonstrated the presence of collagen fibres (blue staining) in the ECM of the control and experimental groups (Figure 2f,h,j). The addition of BMP-7, particularly at 25 ng/mL, enhanced fibroblast proliferation and collagen fiber organization (Figure 2g,h) compared to the control (0 ng/mL) (Figure 2e,f) and 50 ng/mL BMP-7 groups (Figure 2i,j).

**Figure 5:**
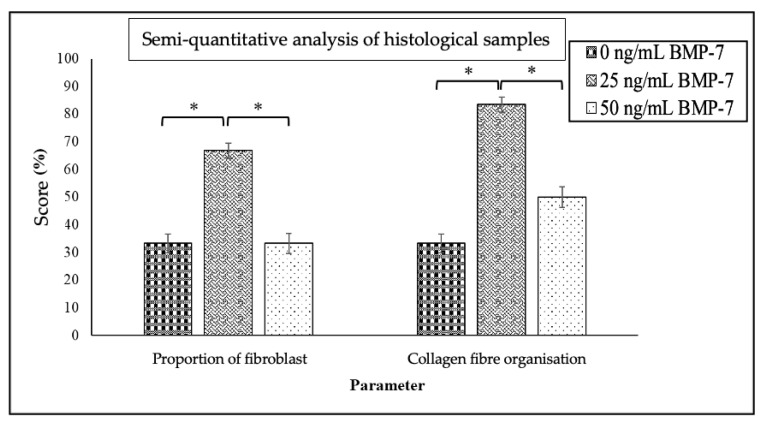
Semi-quantitative analysis based on two parameters: the proportion of fibroblasts present and the organization of collagen fibres through Masson’s trichrome staining of WJMSC on DHDP scaffolds without (0 ng/mL) and with two concentrations of BMP-7 (25 ng/mL and 50 ng/mL) on day 21. Data (*n* = 6) are expressed as mean ± SEM (* *p* < 0.05).

Semi-quantitative analysis of Masson’s Trichrome-stained sections revealed significant differences in the presence of fibroblasts and collagen fiber organization among the groups (Figure 5). The 25 ng/mL BMP-7 group showed a higher proportion of fibroblasts (66.8%) and denser collagen fiber organization (83.4%) compared to the 0 ng/mL (33.2% fibroblasts, 33.2% dense collagen) and 50 ng/mL (33.2% fibroblasts, 50% dense collagen) groups (*p* < 0.05). These results demonstrate that 25 ng/mL BMP-7 has the strongest effect on enhancing fibroblast proliferation and collagen deposition in WJMSCs cultured on DHDP scaffolds.

### 2.5. Proliferation of WJMSCs on DHDP

**Figure 6:**
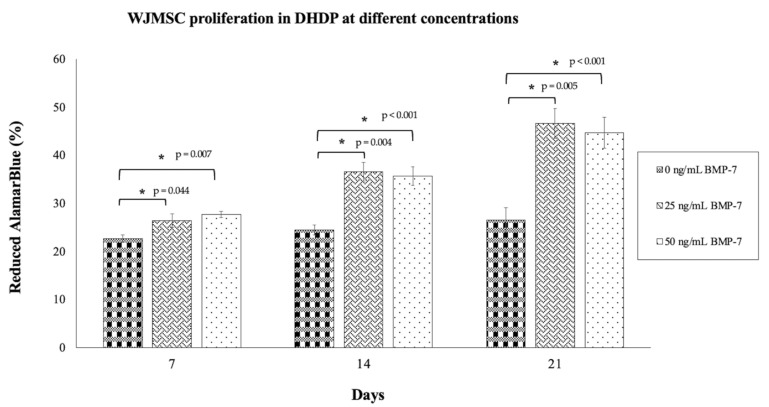
Proliferation of WJMSC in DHDP scaffold without (0 ng/mL BMP-7) and with two concentrations of BMP-7 treatment (25 ng/mL and 50 ng/mL BMP-7), using AlamarBlue readings, wavelengths 570 nm and 600 nm. Data (*n* = 6) are expressed as mean ± SEM. Exact *p* values from the post hoc analysis are displayed on the figure. * *p* < 0.05 indicates a statistically significant difference.

The 25 ng/mL and 50 ng/mL BMP-7 groups showed significantly higher proliferation rates compared to the control group (0 ng/mL BMP-7) on days 7, 14, and 21 (*p* < 0.05) (Figure 6). By day 21, the 25 ng/mL BMP-7 group exhibited the highest proliferation rate (36.62 ± 1.91%), followed by the 50 ng/mL group (35.7 ± 1.92%), while the control group showed the lowest proliferation (26.58 ± 2.62%). These results indicate that BMP-7, particularly at 25 ng/mL and 50 ng/mL, significantly enhances WJMSC proliferation on DHDP scaffolds.

### 2.6. Expression of Dentinogenic Markers in Repopulated DHDP

**Figure 7:**
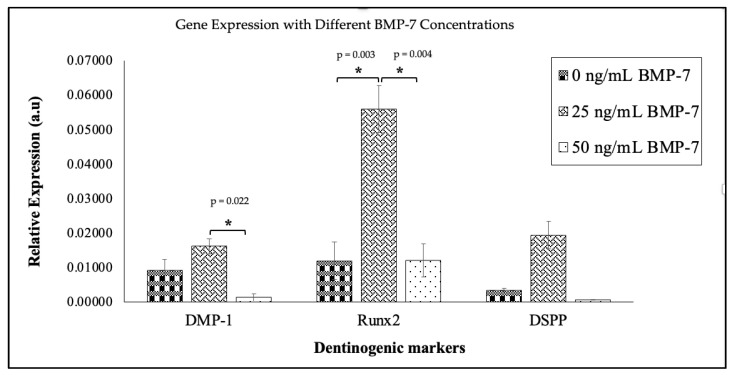
Gene expression levels by relative dentinogenic expression (DMP-1, Runx2, and DSPP) in WJMSC on DHDP scaffolds without (0 ng/mL) and with two concentrations of BMP-7 (25 ng/mL and 50 ng/mL) on day 21. Data (*n* = 6) are expressed as mean ± SEM. Exact *p* values from the post hoc analysis are displayed on the figure. * *p* < 0.05 indicates a statistically significant difference.

The 25 ng/mL BMP-7 group showed significantly higher expression of Runx2 (0.056 ± 0.007) compared to the 50 ng/mL (0.019 ± 0.005) and control (0.016 ± 0.005) groups (*p* < 0.05) at day 21 (Figure 7). Similarly, DMP-1 expression was higher in the 25 ng/mL group (0.012 ± 0.002) compared to the 50 ng/mL group (0.003 ± 0.001). Although DSPP expression did not differ significantly among the groups, the 25 ng/mL BMP-7 group showed a trend toward higher expression (0.012 ± 0.004) compared to the control (0.0013 ± 0.0005) and 50 ng/mL (0.0006 ± 0.0001) groups.

### 2.7. Expression of Dentinogenic Proteins in WJMSCs Cultured on DHDP with and Without BMP-7

Quantifying DSPP and DMP-1 using ELISA involved validating optimized standard curves. For DSPP, serial dilutions were used from 1.57 to 100 ng/mL. This produced a linear regression equation of y = 0.0203x + 0.0495; R^2^ = 0.9866. The DMP-1 standards, with a concentration range of 0.32 to 20 ng/mL, followed by y = 0.0972x + 0.0238 (R^2^ = 0.9876). Both curves showed high linearity, with R^2^ values greater than 0.98, confirming the assay’s precision. The samples were interpolated within the standard range, and the optical density (OD) values for the blanks were recorded. The coefficients of variation (CVs) for both intra- and inter-assays were kept below 10% to ensure reproducibility.

**Figure 8:**
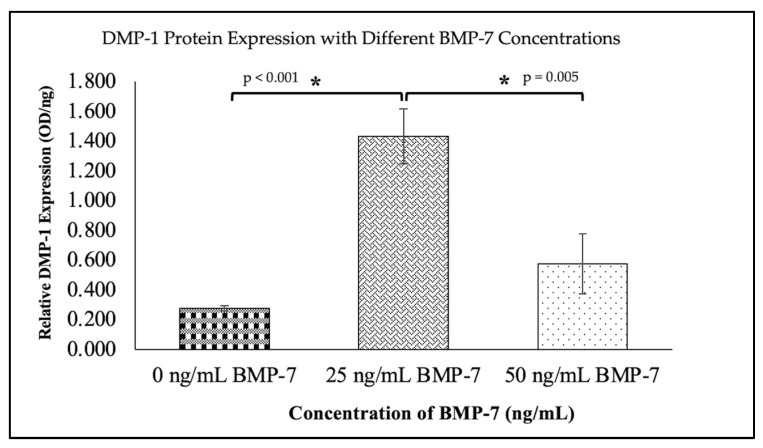
The expression of DMP-1 protein between no BMP-7 treatment (0 ng/mL) and two concentrations of BMP-7 treatment (25 ng/mL and 50 ng/mL) on day 21 using ELISA, with a wavelength of 450 nm. Data (*n* = 6) are expressed as mean ± SEM. Exact *p* values from the post hoc analysis are displayed on the figure. * *p* < 0.05 indicates a statistically significant difference.

**Figure 9:**
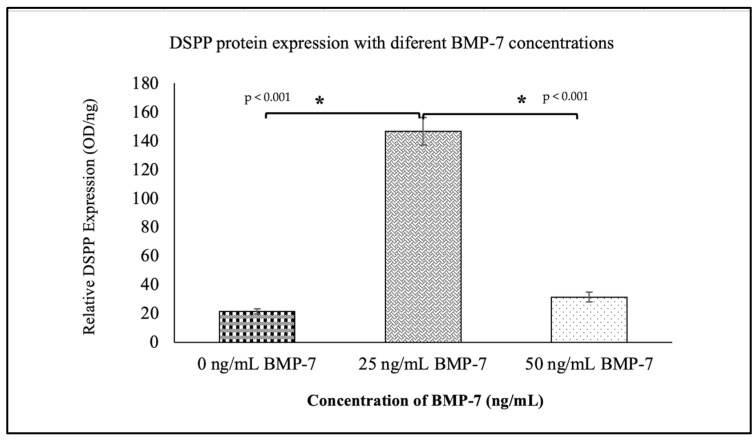
The expression of DSPP protein between no BMP-7 treatment (0 ng/mL) and two concentrations of BMP-7 treatment (25 ng/mL and 50 ng/mL) on day 21 using ELISA, with a wavelength of 450 nm. Data (*n* = 6) are expressed as mean ± SEM. (* *p* < 0.001).

The results of DMP-1 and DSPP protein expression at day 21 are shown in Figure 8 and Figure 9, respectively. The 25 ng/mL BMP-7 group exhibited significantly higher levels of DMP-1 (1.43 ± 0.185) and DSPP (146.7 ± 9.47) compared to the 50 ng/mL and control groups (*p* < 0.05). These findings suggest that 25 ng/mL BMP-7 is the most effective concentration for promoting dentinogenic differentiation of WJMSCs on DHDP scaffolds.

The data and its analysis are available in the Supplementary Materials (Figures S1–S5).

## 3. Discussion

The DHDP scaffolds used in this study were prepared using a previously optimised and validated decellularisation protocol by Song et al. (2017) [24]. The protocol used a hypertonic buffer followed by three cycles of 1% SDS and three cycles of 1% Triton X-100 to remove cellular components from dental pulp tissue while preserving the ECM’s histoarchitecture. The resulting DHDP scaffolds exhibited a porous architecture devoid of cells. The detergent-enzymatic decellularization removes over 95% of cellular components while preserving the original 3D structure of the pulp, including tubular and vascular channels, which are crucial for cell infiltration and nutrient flow [24]. Their scanning electron microscopy confirmed the presence of an interconnected porous network with pore sizes ranging from 50 to 200 µm, which facilitates cell movement and vascularization. Biochemical analyses showed that key ECM proteins, including collagen I, fibronectin, and laminin, were retained at levels exceeding 80%. Additionally, growth factors such as VEGF, FGF-2, and TGF-β1, which support angiogenesis and odontogenic differentiation, remained sequestered within the matrix [27]. All scaffolds included in this study underwent the same standardised preparation process, ensuring consistency and reproducibility across samples. The decellularisation protocol was optimised and validated in a separate work, which confirmed the effective removal of cellular components and the preservation of scaffold architecture. These validated scaffolds were subsequently used consistently throughout this experiment.

The DHDP matrix serves as an effective scaffold for tissue regeneration, preserving the key structural, biochemical, and bioinductive features of the original ECM while closely replicating the natural cellular environment [28]. The potential residual effects of detergents (SDS and Triton X-100) on cell viability and ECM integrity are a concern [29,30]. The evidence of WJMSC proliferation and differentiation in DHDP in our study, however, suggests that the protocol is successful and yields decellularized tissues compatible with the cells. The preserved ECM promotes cell anchorage and regulates cellular activity through its nanostructured environment and network of fibrous and adhesive proteins. The porous architecture of DHDP scaffolds favoured cell infiltration and ECM deposition by WJMSCs. In other studies, the DHDP scaffold has been shown to promote the adhesion and proliferation of DPSCs, resulting in growth over a period of seven days [30]. It also promotes differentiation of these cells, as indicated by increased expression of DSPP, DMP1, and ALP [31]. Co-culturing with endothelial cells (HUVECs) resulted in the formation of capillary-like networks, demonstrating the scaffolds’ ability to promote blood vessel growth. In vivo studies supported its compatibility with living tissue, showing minimal immune response and gradual host-cell infiltration in rat models [32]. Improved mineralization and odontogenic marker expression were demonstrated in DPSCs grown on decellularized pulp ECM and recellularised scaffolds, which led to the formation of vascularized pulp-like tissue in vivo [33]. Together, these studies confirm that decellularized dental pulp is more than just a passive structure; it is a vibrant, bioactive scaffold that replicates the native environment and directs cell behaviour through its structural integrity, retained ECM proteins, and tissue-specific growth factors.

The WJMSCs used in this study exhibited typical MSC characteristics, including adherence to plastic, spindle-shaped morphology, and expression of MSC-positive markers (CD73, CD90, and CD105) while lacking expression of MSC-negative markers (HLA-DRII, CD34, and CD45). These findings align with the criteria established by the International Society for Cellular Therapy (ISCT) for identifying MSCs [34]. However, the low expression of pluripotency markers such as NANOG and SOX2 in WJMSCs at passage 3 suggests reduced pluripotency, consistent with previous studies showing that pluripotency markers decline with increasing passage number [35]. This highlights the importance of using early-passage cells for studies that require high differentiation potential. The ability of WJMSCs to differentiate into adipogenic, osteogenic, and chondrogenic lineages further confirms their multipotency, as outlined by the ISCT. These results are consistent with previous studies demonstrating the differentiation potential of WJMSCs [35]. The successful differentiation of WJMSCs into these lineages supports their suitability for regenerative applications, particularly in dental tissue engineering.

In this study, WJMSCs were characterized at passage 3 to confirm cell identity and purity and subsequently expanded to passage 5 to obtain adequate cell numbers for differentiation assays. Previous studies demonstrate that WJMSCs retain stable biological properties over early passages, including passage 5 [36,37]. Chu et al. (2024) showed high cell viability, preservation of a consistent MSC immunophenotype (CD73^+^/CD90^+^/CD105), and maintenance of multilineage differentiation potential in WJMSCs from passage 1 to passage 5 [36]. These findings indicate that WJMSCs remain biologically stable and suitable for functional assays within this early-passage window. Furthermore, Panwar et al. (2021) [37] assessed long-term in vitro-expanded WJMSCs. They reported no significant differences in the expression of key stemness genes, NANOG and OCT-4 (via qPCR), between passage 2 and passage 6 [37]. Their data support the passage 5 cells maintaining core stemness characteristics required for lineage differentiation. Therefore, the use of passage 5 cells is unlikely to affect their dentinogenic response to BMP-7.

RT-qPCR was used in this study to assess the surface markers (CD73, CD90, CD105) and stemness (pluripotency) markers (NANOG, SOX2, OCT4). While ISCT recommends flow cytometry to validate surface markers [34], quantitative PCR is a reliable alternative for determining MSC identity when flow cytometry is not feasible [38]. Multiple independent reports have shown a strong agreement between mRNA and protein levels for these markers. For example, Abouelnaga et al. (2022) demonstrated that WJMSCs are over 95% CD44^+^, CD90^+^, and CD105^+^ and under 5% CD34^+^ using both flow cytometry and RT-qPCR [39]. Kim et al. (2023) demonstrated mRNA expression (RT-qPCR) and flow cytometry for CD73, CD90, and CD105 in WJ-MSCs, showing similar expression trends [40]. The combination of marker gene expression, morphology, and differentiation behaviour in this study provides sufficient confirmation that the isolated cells were WJMSCs.

Dentinogenesis is governed by a coordinated network of signalling pathways that regulate odontoblast proliferation, differentiation, and matrix secretion. Central to this process are the bone morphogenetic proteins (BMP), constituents of the transforming growth factor-β (TGF-β) superfamily, that drive odontoblast lineage commitment and mineralized matrix formation [41]. TGF-β, released from the dental epithelium and mesenchyme cells during tooth development, activates SMAD1/5/8 dependent transcription to promote extracellular matrix deposition and early odontoblast differentiation [41,42]. The Smad-independent signalling pathway during tooth development and formation also plays an important role in dentinogenesis, with involvement of MAPKs, including major kinases ERK, JNK, and p38, as well as the PI3K/Akt and protein kinase A and C pathways [41,42]. BMP–MAPKs and Smad signalling pathways act synergistically to control dentin development [42]. The coordinated activities of TGF-β, transcriptional factors, and other factors are also essential for dentin development and formation. The Wnt/β-catenin pathway modulates dentine development during repair following pulp damage [43]. Canonical Wnt signalling stabilizes β-catenin to enhance progenitor proliferation, support terminal differentiation, and stimulate reparative dentin formation. These pathways interact extensively—TGF-β can potentiate Wnt activity, and Wnt enhances responsiveness to TGF-β—establishing a regulatory loop essential for normal dentin formation and injury repair [43]. Various BMP ligands are involved in tooth formation, such as BMP-2, BMP-3, BMP-4, BMP-5, BMP-6, BMP-7, and BMP-9. However, only a few of these BMPs have been intensively studied [41]. BMP-7, in particular, is known for its essential role in regulating the differentiation of mesenchymal cells into osteoblasts and, potentially, in bone regeneration [44]. Both in vitro and in vivo animal studies have demonstrated the dentinogenic effect of BMP-7 [45,46,47]. The mechanisms by which BMP-7 promotes the growth and dentin-forming differentiation of WJMSCs involve a complex interplay of Smad-dependent and Smad-independent signalling pathways. This process leads to the activation of important odontogenic markers [41,42,48]. Du et al. (2019) [49] showed that BMP-7 induces phosphorylation of Smad1/5/8 and their subsequent nuclear translocation. This action directly increases the expression of dentin-specific genes such as DSPP and DMP-1 [49]. Meanwhile, activating the p38 MAPK pathway further improves the odontogenic differentiation; blocking either pathway significantly reduces these effects [42,50]. Qin et al. (2016) support these findings in dental pulp stem cells, indicating that BMP7 signalling mechanisms are similar across different mesenchymal stem cell types [50]. Fan et al. (2015) compared BMP-7 to BMP-2, showing that BMP-7 is more effective in activating p38 MAPK and inducing dentinogenic markers [51]. All these findings suggest that BMP-7 is well-suited for dental tissue engineering. Therefore, this growth factor was chosen in this study to investigate its dentinogenic effect on WJMSCs in the DHDP scaffold.

The selection of BMP-7 concentrations (25 and 50 ng/mL) in our study was based on our optimization experiments and prior studies. Previous research has demonstrated that BMP-7 at concentrations of 25–50 ng/mL effectively induces mesenchymal stem cell differentiation [52,53,54]. These findings match our laboratory data, in which 25 and 50 ng/mL BMP-7 consistently promoted WJMSC proliferation without causing cell damage. While a complete dose–response curve could help refine the optimal concentration, the selected doses are well within the biologically active range established in the literature.

Compared with the control (0 ng/mL) group, a significant increase in WJMSC viability was observed at both 25 ng/mL and 50 ng/mL BMP-7 concentrations in this study. This finding reflects well-known receptor-ligand kinetics and feedback systems that support sustained growth responses within this range [55]. Cell viability increases to 50 ng/mL, but there is minimal additional improvement at higher concentrations. This plateau occurs due to basic biological limits in BMP signalling dynamics and negative feedback control [55]. Deschaseaux et al. (2009) demonstrated that BMP-7 reaches receptor saturation in human mesenchymal stem cells [56]. The maximal occupancy of type I/II BMP receptors typically occurs at around 25 ng/mL, which explains why increasing the concentration to 50 ng/mL does not further enhance proliferation. This pattern appears across BMP family members. Langenbach and Handschel, (2007) found similar dose–response plateaus in BMP-2-mediated proliferation due to limited Smad activation beyond receptor saturation [57]. Moreover, BMP signalling activates rapid negative feedback loops via both internal and external mechanisms. Internally, it upregulates inhibitory Smads (Smad6/7), which compete with the receptor for binding and promote receptor degradation [58]. Externally, it induces antagonists, such as noggin, that bind BMP ligands [59,60]. These regulatory processes create the typical bell-shaped dose–response curve seen in many growth factors. Intermediate concentrations, such as 25–50 ng/mL BMP-7 in this case, achieve the highest effects that cannot be surpassed by merely increasing the ligand concentration [61]. These findings explain why both 25 ng/mL and 50 ng/mL BMP-7 result in significantly higher viability than the control, with no significant difference between the two concentrations.

In our study, the percentage of fibroblasts and the arrangement of collagen fibres in the decellularized pulp tissues indicated that WJMSCs thrived well within the scaffolds. Histological analysis revealed that BMP-7, particularly at 25 ng/mL, increased fibroblast proliferation and promoted a denser organization of collagen fibres compared to the control and 50 ng/mL BMP-7 groups. These findings are consistent with previous studies demonstrating that BMP-7 promotes fibroblast proliferation and collagen synthesis across various tissue-engineering contexts [62].

The expression of dentinogenic markers (DSPP, DMP-1, and Runx2) was significantly higher in WJMSCs treated with 25 ng/mL BMP-7 compared to the 50 ng/mL and control groups. Our data showed that 25 ng/mL BMP-7 significantly improves dentinogenic differentiation compared to 0 ng/mL and 50 ng/mL BMP-7. This suggests its effectiveness in promoting WJMSC for pulp regeneration. The increased expression of Runx2, a key transcription factor in odontoblast differentiation, further supports the role of BMP-7 in promoting dentinogenesis [61]. These findings are consistent with previous studies demonstrating that BMP-7 induces odontoblast-like differentiation in DPSCs and other dental stem cells [54,62]. ELISA results confirmed that BMP-7 at 25 ng/mL significantly increased the expression of dentinogenic proteins (DMP-1 and DSPP) in WJMSCs. This further validates the data analysis, which showed that 25 ng/mL BMP-7 caused the greatest increase in dentinogenic markers, including DSPP and DMP-1. This finding supports its use in pulp regeneration.

Dentinogenic gene expression at the mRNA level is documented to be remarkably low during early tooth development. Multiple transcriptomic and validation studies support this finding. Landin et al. (2012) [63] conducted gene expression profiling across mouse tooth development. They demonstrated that dentinogenic and enamel matrix genes, including Ambn, Amelx, Enam, DSPP, DMP1, and COL1A1, exhibit low mRNA levels during prenatal stages (E11.5–P0) [63]. These levels increase significantly only after birth (P1–P7) when odontoblasts and ameloblasts begin to differentiate functionally. These observations were confirmed by both microarray and quantitative RT-PCR, with strong agreement between the methods, reinforcing the reliability of the results. The authors noted that low prenatal mRNA levels were not an error but biologically important, reflecting the timing of dentinogenesis [63,64]. Supporting this, Pascal et al. (2008) noted that mRNA-protein correlation can be weak in undifferentiated or early-stage tissues, even for important structural genes [64]. Together, these studies, backed by various validation methods, biological replicates, and protein-level assessments, confirm that low dentinogenic mRNA expression is a real and expected occurrence during specific developmental periods, rather than a technical issue. The disconnect between DSPP mRNA and protein levels in our study likely reflects key post-transcriptional regulatory processes during dentinogenesis. The data revealed no significant changes in DSPP transcript levels, but a noticeable rise in DSPP protein, indicating strong translational control, which aligns with known mRNA-protein discrepancies in biological systems [65]. Several likely explanations deserve attention. First, microRNA-mediated regulation, as demonstrated by Wan et al. (2014) and Xu et al. (2017), could enable DSPP mRNA to persist while effectively regulating its translation, particularly through miR-21 and miR-221, which directly target DSPP transcripts [66,67]. Second, RNA-binding proteins might stabilize DSPP mRNA or enhance its recruitment to ribosomes without altering overall transcript levels [68]. Third, the relatively long half-life of the DSPP protein, which may exceed 72 h, compared to its mRNA, typically under 24 h, could lead to protein buildup even with temporary transcriptional activity [69,70]. This is especially important in mineralization, where matrix proteins such as DSPP must be continuously present despite fluctuating gene expression. The weak correlation, often below 40%, between mRNA and protein levels across biological systems [71] further indicates that the findings reflect a biologically relevant scenario rather than an experimental error. These post-transcriptional regulatory mechanisms enable cells to rapidly respond to differentiation signals, such as BMP-7, by utilizing existing transcripts for translation while tightly controlling transcription output. This strategy efficiently produces significant amounts of extracellular matrix proteins during dentinogenesis.

Several limitations are acknowledged for this study. Firstly, this study used RT-qPCR to assess MSC surface markers. We recognize that the flow cytometry would have strengthened the phenotypic characterization of the WJMSCs. Secondly, all scaffolds in this study were repopulated with WJMSCs and subsequently treated with BMP-7, with an untreated group serving as the control. Therefore, any observed odontogenic differentiation can be explicitly attributed to BMP-7. However, due to this study design, the potential synergistic interaction between BMP-7 and the DHDP-derived extracellular matrix could not be determined. Thirdly, this study focuses on evaluating the dentinogenic response of WJMSCs to BMP-7 within DHDP scaffolds. Residual DNA quantification, pore morphology characterisation, and mechanical testing are important parameters for confirming decellularisation efficiency and scaffold reproducibility. These assessments were conducted during scaffold optimisation but were not included in the present manuscript, which focuses primarily on the biological outcomes of BMP-7 stimulation. We acknowledge this as a limitation and aim to report these scaffold characterisation data comprehensively in a separate publication.

Despite demonstrating early odontogenic responses, the current study is limited by its short observation period, which restricts conclusions regarding the long-term regenerative capacity of the constructs. Early markers such as DMP-1, Runx2, and DSPP provide only an initial snapshot of lineage commitment and may not reliably predict the formation of functional dentin–pulp–like tissue or sustained mineralization [72]. To address this, future work should incorporate an in vitro mineralization assay to enable quantitative evaluation of the stability and maturation of mineral deposition. Longitudinal profiling of mineralization-related genes at multiple time points, capturing the transition from early regulatory factors to late matrix-mineralizing proteins, will be essential to determine whether the differentiation trajectory follows a genuine maturation sequence, as highlighted in staged mesenchymal stem cell differentiation roadmaps [73]. Mineralisation in cell culture can be induced by supplementing the culture medium with an osteogenic/odontoblast differentiation medium, usually containing β-glycerophosphate (BGP), ascorbic acid (AA), and dexamethasone (DX) [72,73]. The phosphate supplementation provided by BGP enables the chemical reaction for hydroxyapatite formation, and DX steroid stimulates cell differentiation towards a mineralizing lineage via activation of the Wnt/β-catenin signalling pathway [72]. AA is necessary for facilitating the synthesis and secretion of collagen type I, which subsequently provides the scaffold for the mineralised extracellular matrix [72]. Cells are usually maintained in mineralizing media for at least 21 days [72,73]. Cellular mineralization processes can be analysed using experimental approaches such as Von Kossa (VK) stain, Alizarin red S (ARS) stain, and alkaline phosphatase (ALP) activity [72,73]. Furthermore, meaningful validation of regenerative capacity requires long-term in vivo studies capable of assessing functional outcomes such as stable dentin formation, vascularization, and integration with host tissue, similar to the extended evaluations emphasized in biomaterial-guided regeneration research [74]. Collectively, these approaches are necessary to establish whether the early cellular responses observed here ultimately translate into durable, clinically relevant tissue regeneration.

The infection resistance of decellularized scaffolds depends on both the natural properties of the ECM and targeted changes to improve antimicrobial activity. Previous studies indicate that proper decellularization is crucial, as residual cellular debris can attract bacteria and promote biofilm formation. In contrast, well-processed ECM shows better resistance to microbial colonization [75]. Decellularized scaffolds can also influence host immune responses by encouraging an anti-inflammatory M2 macrophage phenotype. This phenotype releases defensins and other antimicrobial peptides, which help control infections [76]. Moreover, the natural composition of ECM may offer basic protection, as some matrices contain hidden antimicrobial peptides that are released during remodelling [77]. Certain decellularized tissues, such as small intestinal submucosa, exhibit natural antibacterial activity against common pathogens, including *Staphylococcus aureus* and *Escherichia coli* [78]. Similarly, it is hypothesized that DHDP would exhibit strong antimicrobial properties while also supporting the regenerative properties needed for clinical use; hence, further evaluation is warranted in future studies.

Although WJMSCs show promising therapeutic potential, their clinical use necessitates careful consideration of potential side effects, informed by current safety data. Clinical trials have generally reported good safety profiles, with most adverse events being mild and temporary. In a phase I trial of allogeneic WJMSC (EN001) infusions for Duchenne muscular dystrophy, typical side effects included local redness and swelling at injection sites, an unusual smell, and headaches, with no dose-limiting toxicities or serious adverse events reported [79]. Similarly, studies of WJMSC therapy for steroid-refractory graft-versus-host disease reported only transient low-grade fevers in some patients, with no major toxicities or long-term complications [80]. However, thorough reviews highlight more serious theoretical risks that warrant attention, including thromboembolic events, pulmonary embolism, transplant-site fibrosis, and rare cases of tumour formation, especially with long-term culture-expanded cells [80]. Encouragingly, longitudinal studies, such as the 24-month follow-up of WJMSC implantation in patients with type 1 diabetes, reported no immediate or long-term side effects [81]. Yet, the authors rightly highlight the need for continuous immunological and metabolic monitoring. Overall, these findings suggest that while WJMSCs have a relatively mild short-term safety profile, careful donor screening, standardized preparation methods, and long-term monitoring are essential to minimize potential risks associated with cellular therapies.

Finally, while this study focused on in vitro experiments, future research should explore the regenerative potential of WJMSCs and BMP-7 on DHDP scaffolds in vivo. One recent study reported the use of DHDP repopulated with WJMSCs for a regenerative endodontic procedure in felines [82]. Histological evaluation revealed the presence of pulp-like tissues in the canals of canine teeth. However, the canals were infection-free [82]. Preclinical studies in animal models could be conducted using infected teeth to mimic the clinical situation to evaluate the safety, efficacy, and outcome of this approach before clinical translation.

## 4. Materials and Methods

### 4.1. Optimization of BMP-7 Concentration (MTT Assay)

Recombinant human BMP-7 (BioLegend, San Diego, CA, USA) was reconstituted in 100 µL of DNase/RNase-free distilled water (dH_2_O) to create a 100 µg/mL stock solution (0.1 µg/µL). This stock was diluted with 1% bovine serum albumin (BSA) to make a 10,000 ng/mL working solution. Both solutions were stored at −20 °C. For cell treatments, the stock was diluted into complete culture medium [Dulbecco’s Modified Eagle medium (DMEM) (Gibco, Thermo Fisher Scientific, Waltham, MA, USA) supplemented with 10% foetal bovine serum (FBS) (Capricorn Scientific, Ebsdorfergrund, Germany), 1% antibiotic/antimycotic (A/A), and 1% L-glutamine (Sigma Aldrich, Saint Louis, MO, USA)], yielding a 100 ng/mL BMP-7 solution. Two-fold serial dilutions (6.25, 12.5, 25, 50 ng/mL) and ten-fold titrations (60, 70, 80, 90 ng/mL) were made from this solution. Each dilution was vortex-mixed thoroughly before being transferred to fresh microcentrifuge tubes.

MTT assays were performed to determine the optimal BMP-7 concentration that promotes cell viability and proliferation in WJMSCs. BMP-7 was tested at concentrations ranging from 0 to 80 ng/mL, with 0 ng/mL as the control. Based on the MTT assay findings, 25 ng/mL and 50 ng/mL BMP-7 were selected for subsequent experiments involving WJMSCs cultured on decellularized pulp scaffolds. These concentrations were chosen as 25 ng/mL yielded the highest cell viability, while 50 ng/mL showed the second-highest viability among the tested doses (Figure 1).

### 4.2. Decellularization of Human Dental Pulp

**Figure 10:**
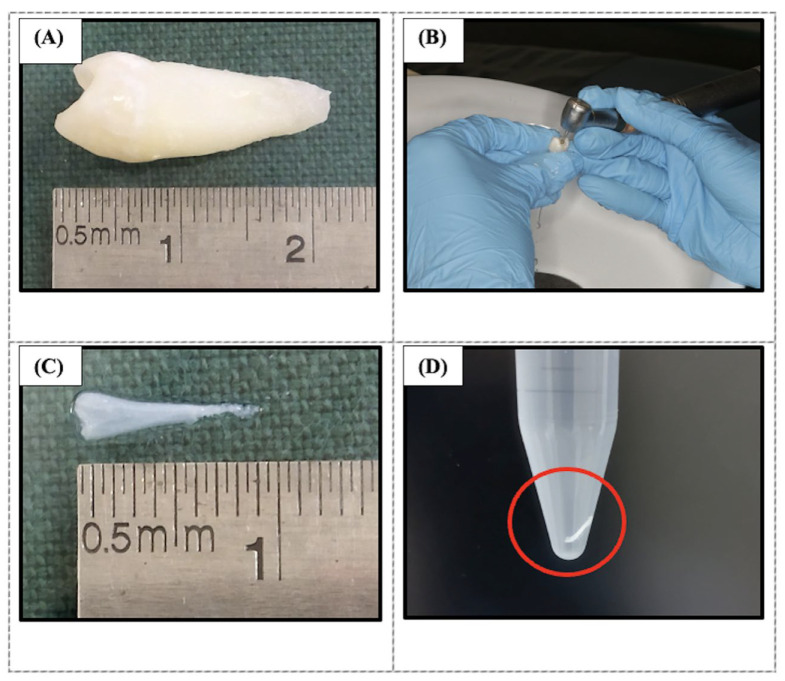
Photographs showing the extraction of pulp tissue. (**A**) The collected permanent tooth, measuring 2.3 mm. (**B**) The pulp tissue was extracted from the tooth following the drilling procedure and removed using K files. (**C**) The pulp tissue, measuring 0.9 mm. (**D**) The pulp tissue (red circle) was rinsed with PBS + 1% A/A before decellularization.

Human dental pulps were obtained from extracted sound premolar or molar teeth of patients aged 18–40 years [83], following ethical approval (Reference Number: UKM PPI/111/8/JEP-2023-318) and informed consent. The teeth collected were non-cavitated and extracted for orthodontic purposes. Access to the pulp chamber was obtained, and the pulp tissues were extirpated en masse (Figure 10). The decellularization protocol was adapted from Song et al. (2017), with modifications from Traphagen et al. (2012) and Datta et al. (2021), as outlined in Table 1 [24,84,85]. The pulp tissue was decellularized using Tris-HCl buffer, 1% sodium dodecyl sulfate (SDS) (Bendosen, Damansara, Malaysia), and 1% Triton X-100 (Vivantis, Subang Jaya, Malaysia). The decellularized tissues were washed with phosphate-buffered saline (PBS) containing 1% A/A and stored at −20 °C until further use. The samples were fixed with 4% paraformaldehyde (Merck, Darmstadt, Germany), embedded in paraffin (Surgipath, Leica, Nussloch, Germany), sectioned at a thickness of 4–5 µm, and stained with hematoxylin (Sigma-Aldrich, Saint Louis, MO, USA) and eosin (Surgipath, Leica, Nussloch, Germany) (H&E) and Masson’s Trichrome (Solar Bio, Beijing, China) to evaluate the decellularization process. Samples were observed under a light microscope (Eclipse Nikon Ni-U, Tokyo, Japan).

### 4.3. Wharton’s Jelly Mesenchymal Stem Cells Isolation and Culture

**Figure 11:**
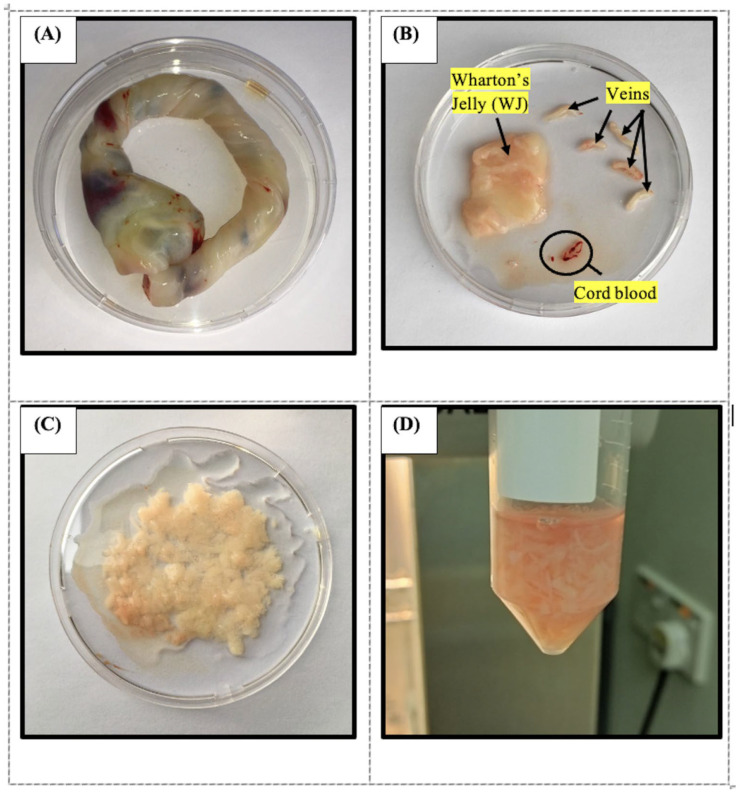
Photographs showing the process of isolating Wharton’s Jelly (WJ) from the umbilical cord and culture of WJMSC. (**A**) The collected human umbilical cord. (**B**) The umbilical vein and umbilical cord blood were removed from the umbilical cord. (**C**) The WJ is rinsed with PBS and then finely chopped. (**D**) WJ fragments were digested using 0.3% type I collagenase.

WJMSCs were isolated from umbilical cords obtained from healthy pregnant women aged 18–40 years who delivered full-term babies (>39 weeks) via caesarean section [86]. With ethical approval, the umbilical cords were collected from the Hospital Canselor Tuanku Muhriz (HCTM), Universiti Kebangsaan Malaysia (UKM). The umbilical cords were collected in a sterile collection bottle containing PBS (SolarBio, Beijing, China) within 24 h following the delivery. The umbilical cords were processed as soon as possible, using multiple washes with sterile PBS and 1% A/A (10,000 units/mL of penicillin, 10,000 μg/mL of streptomycin, and 25 μg/mL of Amphotericin B) (Gibco, Thermo Fisher Scientific, Waltham, MA, USA). The cords were processed to isolate Wharton’s Jelly, which was then digested using 0.3% collagenase type I (Worthington Biochemical Corporation, Lakewood, NJ, USA) (Figure 11). The isolated cells were cultured in a complete Dulbecco’s Modified Eagle medium (DMEM) (Gibco, Thermo Fisher Scientific, Waltham, MA, USA) supplemented with 10% foetal bovine serum (FBS) (Capricorn Scientific, Ebsdorfergrund, Germany), 1% A/A, and 1% L-glutamine (Sigma Aldrich, Saint Louis, MO, USA) at 37 °C in a 5% CO_2_ humidified incubator. Cells were passaged when they reached 80–90% confluence and used for experiments at passage 5.

### 4.4. Characterization of WJMSCs

Cell morphology was observed under an inverted microscope. The differentiation of WJMSCs into adipogenic, osteogenic, and chondrogenic lineages was investigated, followed by genetic analysis. For adipogenic differentiation, WJMSCs were seeded at 5 × 10^4^ cells/cm^3^ in 6-well plates and cultured in adipogenic differentiation medium (ThermoFisher, Waltham, MA, USA, Catalog No: A10070-01) for 14 days. Lipid accumulation was assessed using Oil Red O staining (ScienCell, Carlsbad, CA, USA). For osteogenic differentiation, cells were seeded at 1 × 10^5^ cells/cm^3^ and cultured in an osteogenic medium (Thermo Fisher Scientific, Waltham, MA, USA, Catalog No. A10072-01) for 28 days. Calcium deposition was visualized using Alizarin Red S staining. WJMSCs were pelleted at 1.6 × 10^7^ cells/cm^3^ for chondrogenic differentiation and cultured in a chondrogenic medium (ThermoFisher, USA, Catalog No: A10071-01) for 21 days. Sulfated glycosaminoglycans (sGAGs) were detected using Alcian Blue and Nuclear Fast Red staining (ScienCell, Carlsbad, CA, USA). All stained samples were imaged using an inverted microscope, with images captured at 10× and 20× magnification.

Specific MSC markers were selected and analysed to confirm WJMSCs’ stem cell properties. WJMSCs were cultured in T25 flasks at passage 3, with a seeding density of 1 × 10^6^ cells/cm^3^. The cells were maintained in a complete culture medium under standard conditions (37 °C, 5% CO_2_) until they reached 80–90% confluence. The expression of MSC markers, including positive markers (CD73, CD90, CD105) and negative markers (HLA-DR, CD34, CD45), and pluripotency markers (OCT-4, NANOG, SOX2), was assessed using reverse transcriptase-quantitative polymerase chain reaction (RT-qPCR) to confirm differentiation, as listed in Table 2.

### 4.5. Repopulation of Decellularized Pulp Tissues with WJMSC

The total volume of the decellularized scaffold for each group was set. The scaffolds were selected for WJMSC seeding and washed with PBS + 1% A/A. Three experimental groups were established: (1) Control (0 ng/mL BMP-7), (2) 25 ng/mL BMP-7, and (3) 50 ng/mL BMP-7. Each group consists of six samples (*n* = 6).

The desired cell density for each scaffold was 5 × 10^4^. Cell count was performed using a haemocytometer using the Trypan Blue Exclusion Method. Then, the cell suspension was placed on the haemocytometer until it filled the cell-counting grid. The haemocytometer was viewed under an inverted microscope. Cell counting involved only transparent cells representing live cells, while dark blue cells, which were dead, were excluded.

WJMSCs were centrifuged for 10 min at 4 °C at a speed of 600× *g*. The supernatant was discarded, and the pellet was suspended in 20 µL of DMEM complete media, which was used to seed WJMSCs onto each scaffold in a 96-well plate. The repopulated DHDPs were incubated for 60 min. Complete media DMEM, without and with BMP-7 (25 ng/mL and 50 ng/mL), was added to the respective wells. WJMSCs were cultured at 37 °C in 5% CO_2_. Cell proliferation was assessed on days 7, 14, and 21, while histological, gene, and protein expression analyses were performed on day 21.

### 4.6. Cell Proliferation Assay

Cell proliferation was measured using the AlamarBlue assay. Repopulated DHDP (days 7, 14, and 21 post-treatment) were incubated with AlamarBlue reagent (Thermo Fisher Scientific, Waltham, MA, USA) for 4 h at 37 °C. The absorbance was measured at 570 nm and 600 nm using a microplate reader. The percentage reduction in AlamarBlue was calculated using the formula provided in the AlamarBlue Assay Thermo Fisher Scientific manual, and the results were analysed to determine cell viability and proliferation.

### 4.7. Histological and Semi-Quantitative Analysis

On day 21, the repopulated samples were fixed in 10% formalin overnight. The fixed tissues were processed using an automated tissue processor. The tissues were dehydrated, cleared, and embedded in paraffin wax (Leica, Düsseldorf, Germany). Sections 4–5 µm thickness were cut using a microtome and mounted on glass slides. The tissue sections were stained with H&E and Masson’s Trichrome (Solarbio, Beijing, China) according to the manufacturer’s protocols. H&E staining was used to visualize general tissue morphology, while Masson’s Trichrome staining was employed to assess collagen fiber organization. The stained sections were examined under a light microscope, and images were captured at 10× magnification.

Semi-quantitative analysis was performed for all Masson’s Trichrome-stained sections [87,88] using a scoring system to evaluate two parameters: the proportion of fibroblasts and the arrangement of collagen fibres. Each sample was graded using two score scales:

For the proportion of fibroblasts:

Score 0: No fibroblasts;
Score 1: Presence of fibroblasts (spindle-shaped cells).

For the arrangement of collagen fibres:

Score 0: Loose arrangement of collagen fibres (loosely arranged and interwoven in all directions);
Score 1: Dense (clearly arranged with collagen fibres forming collagen lamellae) [88].

Two raters (NIMA) and (NAAS) scored the samples. The raters underwent training and calibration for the evaluations. Six tissue samples for each group were rated twice by both raters using the scoring scale. The scoring was performed independently. Intra-rater and inter-rater reliability were assessed using Cohen’s kappa coefficient test. If the two raters disagreed on the scoring of the samples, a discussion was held to reach consensus on the final score. The inter-rater Kappa values showed fair agreement (κ = 0.04), and intra-rater Kappa values indicated moderate agreement, respectively (Rater 1: κ = 0.54; Rater 2: κ = 0.6).

### 4.8. Gene Expression Analysis

Total RNA was extracted from repopulated DHDP using TRIzol reagent (Thermo Fisher Scientific, USA). The RNA concentration and purity were determined using a Nanodrop spectrophotometer. cDNA was synthesized from 100 ng total RNA for each group using the SensiFAST cDNA Synthesis Kit (Bioline, UK). Gene expression levels of dentinogenic markers (DSPP, DMP-1, and Runx2) were quantified using RT-qPCR, as the primers listed in Table 3. The reactions were performed using the SensiFAST SYBR No-ROX Kit (Bioline, UK) on a real-time PCR system. Relative gene expression was calculated using glyceraldehyde-3-phosphate dehydrogenase (GAPDH) as the housekeeping gene.

The results were calculated with the following formula and analysed to determine dentinogenic activity in WJMSCs;

$$\mathrm{Relative}\;\mathrm{gene}\;\mathrm{expression}=2^{-[\Delta Cq\;sample-\Delta Cq\;control]}$$

ΔCq control = Data quantification reading GADPH
ΔCq sample = Data quantification reading of each primer (DSPP, DMP-1, and Runx2)

Quantitative PCR was performed using SYBR Green Master Mix (Bioline, London, UK) on a CFX96 Touch Real-Time PCR Detection System (Bio-Rad, Hercules, CA, USA). The primer efficiencies were confirmed (98–102%) through standard curves made from 5-point 10-fold dilutions of cDNA. Melt curves demonstrated single amplicons. For relative quantification, the ΔΔCt method was applied with normalization to GAPDH [89]. The assays followed MIQE guidelines [90].

### 4.9. Protein Expression Analysis

Protein expression of DSPP and DMP-1 was analysed using enzyme-linked immunosorbent assay (ELISA) (ELK Biotechnology, Wuhan, China). The assays were performed according to the manufacturer’s instructions. The samples were incubated in 96-well plates coated with specific antibodies. After a series of washes and incubations with biotinylated antibodies and streptavidin-horseradish peroxidase, the reaction was developed using a tetramethylbenzidine substrate. The absorbance was measured at 450 nm, and the protein concentration was determined using a standard curve.

### 4.10. Statistical Analysis

All statistical analyses were performed using SPSS version 27. The normality of the data was assessed using the Shapiro–Wilk test, and the homogeneity of variances was confirmed using Levene’s test. For comparisons between groups, one-way ANOVA was performed followed by post hoc tests: the Tukey test was used for MTT assay (cell viability with BMP-7 treatments), AlamarBlue assay (cell proliferation on repopulated DHDP), and ELISA for DMP-1, while the Games–Howell test was applied for quantitative PCR analyses (DMP-1, Runx2, DSPP) and ELISA for DSPP. For the semi-quantitative analysis, the Kruskal–Wallis test was used. Data were presented as mean ± standard error of the mean (SEM), and a *p*-value of <0.05 was considered statistically significant.

## 5. Conclusions

This study demonstrates that BMP-7, particularly at 25 ng/mL, significantly enhances the proliferation, differentiation, and dentinogenic potential of WJMSCs cultured on DHDP scaffolds. The findings suggest that BMP-7 at 25 ng/mL promotes dental pulp regeneration, as evidenced by increased fibroblast proliferation, collagen fiber organization, and expression of dentinogenic markers. These results highlight the potential of WJMSCs and BMP-7 for regenerative dentistry and provide a foundation for future studies to optimize scaffold design and evaluate the approach for clinical application. 

## Figures and Tables

**Table 1:** Protocol for decellularization of pulp tissue.

Chemical Solution	Time (h)	Cycle		References
Tris-HCl buffer	48	1		Traphagen et al. (2012) [84]
1% SDS 1% Triton X-100	24	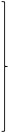	3	Song et al. (2017) [24]
	24			
1% SDS 1% Triton X-100	24			
	24			
1% SDS 1% Triton X-100	24			
	24			
PBS + 1% A/A	24	1		Datta et al. (2021) [85]

**Table 2:** List of MSC markers used for characterization of WJMSC.

Type of Markers	Gene	Primer Sequences (5′–3′) [Forward, F] [Reverse, R]	Fragment Length (bp)	Accession Number
Housekeeping	*GAPDH*	F: CAATGACCCCTTCATTGACC R: TTGATTTTGGAGGGATCTCG	160	NM_002046.5
Pluripotent	*NANOG*	F: TCCTCCTGCCTGAGTCTCTC R: ATACAGGGCTAGGCTGGTGA	199	NM_024865
	*OCT-4*	F: GCAAAGCAGAAACCCTCGTG R: AACCACACTCGGACCACATC	172	NM_002701
	*SOX2*	F: ATGGGTTCGGTGGTCAAGTC R: ACATGTGAAGTCTGCTGGGG	166	NM_003106
MSC Positive	*CD73*	F: CCAGCAGTTGAAGGTCGGAT R: CTGTCACAAAGCCAGGTCCT	196	NM_002526
	*CD90*	F: TGGTGAGAAGAGCTGCTGTG R: CACACAGTGCCGCTCATTTC	122	NM_006288
	*CD105*	F: CCTACGTGTCCTGGCTCATC R: CGAAGGATGCCACAATGCTG	174	NM_000118
MSC Negative	*HLA-DR II*	F: GTCAATGTCACGTGGCTTCG R: TCCACCCTGCAGTCGTAAAC	149	NM_019111.5
	*CD34*	F: CTCAGCTCAATCGCCTCCAT R: CAAGCCACCTCCCTTCTCTG	191	NM_001025109
	*CD45*	F: ATGATTGCTGCTCAGGGACC R: TCTCCCCAGTACTGAGCACA	140	NM_002838

**Table 3:** List of dentinogenic markers, including a housekeeping gene for gene expression.

Gene	Primer Sequences (5′–3′) [Forward, F] [Reverse, R]	Fragment Length (bp)	Accession Number
*GAPDH*	F: CAATGACCCCTTCATTGACC R: TTGATTTTGGAGGGATCTCG	160	NM_002046.5
*DSPP*	F: GACACCCAGAAGCTCAACCA R: CACTGCTGGGACCCTTGATT	124	NM_014208.3
*DMP-1*	F: GCACACACTCTCCCACTCAA R: CTCGCTCTGACTCTCTGCTG	169	NM_004407.4
*Runx2*	F: CTGTGGCATGCACTTTGACC R: CTTGGGTGGGTGGAGGATTC	161	NM_001024630.4

## Data Availability

The raw data supporting the conclusions of this article will be made available by the authors on request.

## Supplementary Materials

The following supporting information can be downloaded at: https://www.mdpi.com/article/10.3390/ijms262311760/s1.

## Abbreviations

The following abbreviations are used in this manuscript:

BMP-7	Bone morphogenetic protein 7
DHDP	Decellularized human dental pulp
DPSC	Dental pulp stem cell
DSPP	Dentin sialophosphoprotein
DMP-1	Dentin matrix acidic phosphoprotein 1
GADPH	Glyceraldehyde-3-phosphate dehydrogenase
ECM	Extracellular matrix
GDM	Gestational diabetes mellitus;
MSC	Mesenchymal stem cell
RET	Regenerative endodontic therapy
RCT	Root canal treatment
SDS	Sodium dodecyl sulfate
VEGF	Vascular endothelial growth factor
WJMSC	Wharton’s jelly mesenchymal stem cell
