# Supplementary material for: Dentinogenic Effect of BMP-7 on Wharton’s Jelly Mesenchymal Stem Cells Cultured in Decellularized Dental Pulp
Source: Int J Mol Sci. 2025 Dec 4;26(23):11760. doi: 10.3390/ijms262311760 (PMC12693673; doi:10.3390/ijms262311760)
Supplement: Supplementary file 1 [file ijms-26-11760-s001.zip › ijms-3988969-supplementary.pdf]

Dentinogenic Effect of BMP-7 on Wharton's Jelly Mesenchymal Stem Cells Cultured in Decellularized Dental Pulp

Nur Athirah Ahmad Shuhaimi [1], Dalia Abdullah [1], Farinawati Yazid [2,*], Sook Luan Ng [3,*], Nurul Inaas Mahamad Apandi [3] and Nur Azurah Abdul Ghani [4]

1 Department of Restorative Dentistry, Faculty of Dentistry, Universiti Kebangsaan Malaysia, Kuala Lumpur 50300, Malaysia; p131245@siswa.ukm.edu.my (N.A.A.S.); daliaabdullah@ukm.edu.my (D.A.)

2 Department of Family Oral Health, Faculty of Dentistry, Universiti Kebangsaan Malaysia, Kuala Lumpur 50300, Malaysia

3 Department of Craniofacial Diagnostics and Biosciences, Faculty of Dentistry, Universiti Kebangsaan Malaysia, Kuala Lumpur 50300, Malaysia; nurulinaas@ukm.edu.my

4 Department of Obstetrics and Gynecology, Faculty of Medicine, Universiti Kebangsaan Malaysia, Jalan Yaakob Latif, Bandar Tun Razak, Kuala Lumpur 56000, Malaysia

* Correspondence: drfarinawati@ukm.edu.my (F.Y.); ngsookluan@ukm.edu.my (S.L.N.); Tel.: +60-392-897-076 (F.Y.); +60-392-897-997 (S.L.N.)

Tests of Normality

	BMP7concentrations	Kolmogorov-Smirnov[a] Statistic	df	Sig.	Shapiro-Wilk Statistic	df	Sig.
CellViability_MTT	0ng/ml BMP-7	.	6	.	.	6	.
	6.25ng/ml BMP-7	.317	6	.060	.888	6	.307
	12.5ng/ml BMP-7	.191	6	.200*	.952	6	.757
	25ng/ml BMP-7	.192	6	.200*	.927	6	.558
	50ng/ml BMP-7	.285	6	.138	.898	6	.362
	60ng/ml BMP-7	.273	6	.183	.848	6	.151
	70ng/ml BMP-7	.230	6	.200*	.883	6	.282
	80ng/ml BMP-7	.218	6	.200*	.933	6	.600

*. This is a lower bound of the true significance.

a. Lilliefors Significance Correction

(a)

Tests of Homogeneity of Variances

		Levene Statistic	df1	df2	Sig.
CellViability_MTT	Based on Mean	2.315	7	40	.044
	Based on Median	2.016	7	40	.077
	Based on Median and with adjusted df	2.016	7	21.468	.100
	Based on trimmed mean	2.366	7	40	.040

(b)

ANOVA

CellViability_MTT

	Sum of Squares	df	Mean Square	F	Sig.
Between Groups	9988.690	7	1426.956	140.417	<.001
Within Groups	406.489	40	10.162		
Total	10395.179	47			

(c)

Multiple Comparisons

Dependent Variable: CellViability_MTT

	(I) BMP7concentrations	(J) BMP7concentrations	Mean Difference (I-J)	Std. Error	Sig.	95% Confidence Interval	
						Lower Bound	Upper Bound
Tukey HSD	0ng/ml BMP-7	6.25ng/ml BMP-7	-9.09333*	1.84049	<.001	-14.9765	-3.2102
		12.5ng/ml BMP-7	-19.27667*	1.84049	<.001	-25.1598	-13.3935
		25ng/ml BMP-7	-50.23000*	1.84049	<.001	-56.1131	-44.3469
		50ng/ml BMP-7	-27.50167*	1.84049	<.001	-33.3848	-21.6185
		60ng/ml BMP-7	-21.69833*	1.84049	<.001	-27.5815	-15.8152
		70ng/ml BMP-7	-14.90500*	1.84049	<.001	-20.7881	-9.0219
		80ng/ml BMP-7	-7.40833*	1.84049	.005	-13.2915	-1.5252
	6.25ng/ml BMP-7	0ng/ml BMP-7	9.09333*	1.84049	<.001	3.2102	14.9765
		12.5ng/ml BMP-7	-10.18333*	1.84049	<.001	-16.0665	-4.3002
		25ng/ml BMP-7	-41.13667*	1.84049	<.001	-47.0198	-35.2535
		50ng/ml BMP-7	-18.40833*	1.84049	<.001	-24.2915	-12.5252
		60ng/ml BMP-7	-12.60500*	1.84049	<.001	-18.4881	-6.7219
		70ng/ml BMP-7	-5.81167	1.84049	.055	-11.6948	.0715
		80ng/ml BMP-7	1.68500	1.84049	.983	-4.1981	7.5681
	12.5ng/ml BMP-7	0ng/ml BMP-7	19.27667*	1.84049	<.001	13.3935	25.1598
		6.25ng/ml BMP-7	10.18333*	1.84049	<.001	4.3002	16.0665
		25ng/ml BMP-7	-30.95333*	1.84049	<.001	-36.8365	-25.0702
		50ng/ml BMP-7	-8.22500*	1.84049	.001	-14.1081	-2.3419
		60ng/ml BMP-7	-2.42167	1.84049	.887	-8.3048	3.4615
		70ng/ml BMP-7	4.37167	1.84049	.281	-1.5115	10.2548
		80ng/ml BMP-7	11.86833*	1.84049	<.001	5.9852	17.7515
	25ng/ml BMP-7	0ng/ml BMP-7	50.23000*	1.84049	<.001	44.3469	56.1131
		6.25ng/ml BMP-7	41.13667*	1.84049	<.001	35.2535	47.0198
		12.5ng/ml BMP-7	30.95333*	1.84049	<.001	25.0702	36.8365
		50ng/ml BMP-7	22.72833*	1.84049	<.001	16.8452	28.6115
		60ng/ml BMP-7	28.53167*	1.84049	<.001	22.6485	34.4148
		70ng/ml BMP-7	35.32500*	1.84049	<.001	29.4419	41.2081
		80ng/ml BMP-7	42.82167*	1.84049	<.001	36.9385	48.7048
	50ng/ml BMP-7	0ng/ml BMP-7	27.50167*	1.84049	<.001	21.6185	33.3848
		6.25ng/ml BMP-7	18.40833*	1.84049	<.001	12.5252	24.2915
		12.5ng/ml BMP-7	8.22500*	1.84049	.001	2.3419	14.1081
		25ng/ml BMP-7	-22.72833*	1.84049	<.001	-28.6115	-16.8452
		60ng/ml BMP-7	5.80333	1.84049	.056	-.0798	11.6865
		70ng/ml BMP-7	12.59667*	1.84049	<.001	6.7135	18.4798
		80ng/ml BMP-7	20.09333*	1.84049	<.001	14.2102	25.9765
	60ng/ml BMP-7	0ng/ml BMP-7	21.69833*	1.84049	<.001	15.8152	27.5815
		6.25ng/ml BMP-7	12.60500*	1.84049	<.001	6.7219	18.4881
		12.5ng/ml BMP-7	2.42167	1.84049	.887	-3.4615	8.3048
		25ng/ml BMP-7	-28.53167*	1.84049	<.001	-34.4148	-22.6485
		50ng/ml BMP-7	-5.80333	1.84049	.056	-11.6865	.0798
		70ng/ml BMP-7	6.79333*	1.84049	.014	.9102	12.6765
		80ng/ml BMP-7	14.29000*	1.84049	<.001	8.4069	20.1731
	70ng/ml BMP-7	0ng/ml BMP-7	14.90500*	1.84049	<.001	9.0219	20.7881
		6.25ng/ml BMP-7	5.81167	1.84049	.055	-.0715	11.6948
		12.5ng/ml BMP-7	-4.37167	1.84049	.281	-10.2548	1.5115
		25ng/ml BMP-7	-35.32500*	1.84049	<.001	-41.2081	-29.4419
		50ng/ml BMP-7	-12.59667*	1.84049	<.001	-18.4798	-6.7135
		60ng/ml BMP-7	-6.79333*	1.84049	.014	-12.6765	-.9102
		80ng/ml BMP-7	7.49667*	1.84049	.005	1.6135	13.3798
	80ng/ml BMP-7	0ng/ml BMP-7	7.40833*	1.84049	.005	1.5252	13.2915
		6.25ng/ml BMP-7	-1.68500	1.84049	.983	-7.5681	4.1981
		12.5ng/ml BMP-7	-11.86833*	1.84049	<.001	-17.7515	-5.9852
		25ng/ml BMP-7	-42.82167*	1.84049	<.001	-48.7048	-36.9385
		50ng/ml BMP-7	-20.09333*	1.84049	<.001	-25.9765	-14.2102
		60ng/ml BMP-7	-14.29000*	1.84049	<.001	-20.1731	-8.4069
		70ng/ml BMP-7	-7.49667*	1.84049	.005	-13.3798	-1.6135
Dunnett t (2-sided)[b]	6.25ng/ml BMP-7	0ng/ml BMP-7	9.09333*	1.84049	<.001	4.0671	14.1196
	12.5ng/ml BMP-7	0ng/ml BMP-7	19.27667*	1.84049	<.001	14.2504	24.3029
	25ng/ml BMP-7	0ng/ml BMP-7	50.23000*	1.84049	<.001	45.2037	55.2563
	50ng/ml BMP-7	0ng/ml BMP-7	27.50167*	1.84049	<.001	22.4754	32.5279
	60ng/ml BMP-7	0ng/ml BMP-7	21.69833*	1.84049	<.001	16.6721	26.7246
	70ng/ml BMP-7	0ng/ml BMP-7	14.90500*	1.84049	<.001	9.8787	19.9313
	80ng/ml BMP-7	0ng/ml BMP-7	7.40833*	1.84049	.002	2.3821	12.4346

*. The mean difference is significant at the 0.05 level.

b. Dunnett t-tests treat one group as a control, and compare all other groups against it.

(d)

Figure S1. Statistical analysis of WJMSC cell viability following treatment with different concentrations of BMP-7: (a) normality assessment using the Shapiro–Wilk test, (b) homogeneity of variances using Levene's test, (c) one-way ANOVA, and (d) post hoc analyses (Dunnett and Tukey tests). The data were normally distributed ($p > 0.05$) and exhibited homogeneous variances ($p > 0.05$). One-way ANOVA revealed a significant difference among the treatment groups ($p < 0.05$). Dunnett's post hoc test showed that all BMP-7–treated groups had significantly higher cell viability compared with the control (0ng/mL BMP-7) ($p < 0.05$). Tukey's post hoc analysis further demonstrated that 25ng/mL BMP-7 produced the highest cell viability, with a significant increase compared to all other concentrations ($p < 0.001$).

Tests of Normality

		Kolmogorov-Smirnov[a]			Shapiro-Wilk		
	Group	Statistic	df	Sig.	Statistic	df	Sig.
Day_7	0 ng/ml BMP-7	.214	6	.200*	.898	6	.360
	25 ng/ml BMP-7	.208	6	.200*	.934	6	.608
	50 ng/ml BMP-7	.169	6	.200*	.960	6	.821
Day_14	0 ng/ml BMP-7	.290	6	.125	.795	6	.054
	25 ng/ml BMP-7	.149	6	.200*	.963	6	.846
	50 ng/ml BMP-7	.211	6	.200*	.870	6	.226
Day_21	0 ng/ml BMP-7	.178	6	.200*	.964	6	.847
	25 ng/ml BMP-7	.234	6	.200*	.870	6	.228
	50 ng/ml BMP-7	.254	6	.200*	.862	6	.195

*. This is a lower bound of the true significance.

a. Lilliefors Significance Correction

(a)

Tests of Homogeneity of Variances

		Levene Statistic	df1	df2	Sig.
Day_7	Based on Mean	1.875	2	15	.188
	Based on Median	1.728	2	15	.211
	Based on Median and with adjusted df	1.728	2	9.798	.228
	Based on trimmed mean	1.869	2	15	.188
Day_14	Based on Mean	.568	2	15	.578
	Based on Median	.594	2	15	.564
	Based on Median and with adjusted df	.594	2	13.033	.566
	Based on trimmed mean	.538	2	15	.595
Day_21	Based on Mean	.962	2	15	.405
	Based on Median	.882	2	15	.435
	Based on Median and with adjusted df	.882	2	12.269	.439
	Based on trimmed mean	.979	2	15	.398

(b)

ANOVA

		Sum of Squares	df	Mean Square	F	Sig.
Day_7	Between Groups	81.432	2	40.716	7.030	.007
	Within Groups	86.870	15	5.791		
	Total	168.302	17			
Day_14	Between Groups	428.464	2	214.232	12.654	<.001
	Within Groups	253.958	15	16.931		
	Total	682.423	17			
Day_21	Between Groups	1495.679	2	747.840	13.882	<.001
	Within Groups	808.047	15	53.870		
	Total	2303.726	17			

(c)

Post Hoc Tests

Multiple Comparisons

Tukey HSD

Dependent Variable	(I) Group	(J) Group	Mean Difference (I-J)	Std. Error	Sig.	95% Confidence Interval Lower Bound	95% Confidence Interval Upper Bound
Day_7	0 ng/ml BMP-7	25 ng/ml BMP-7	-3.702333*	1.389404	.044	-7.31127	-.09340
		50 ng/ml BMP-7	-5.025667*	1.389404	.007	-8.63460	-1.41673
	25 ng/ml BMP-7	0 ng/ml BMP-7	3.702333*	1.389404	.044	.09340	7.31127
		50 ng/ml BMP-7	-1.323333	1.389404	.617	-4.93227	2.28560
	50 ng/ml BMP-7	0 ng/ml BMP-7	5.025667*	1.389404	.007	1.41673	8.63460
		25 ng/ml BMP-7	1.323333	1.389404	.617	-2.28560	4.93227
Day_14	0 ng/ml BMP-7	25 ng/ml BMP-7	-9.196500*	2.375610	.004	-15.36708	-3.02592
		50 ng/ml BMP-7	-11.207667*	2.375610	<.001	-17.37824	-5.03709
	25 ng/ml BMP-7	0 ng/ml BMP-7	9.196500*	2.375610	.004	3.02592	15.36708
		50 ng/ml BMP-7	-2.011167	2.375610	.681	-8.18174	4.15941
	50 ng/ml BMP-7	0 ng/ml BMP-7	11.207667*	2.375610	<.001	5.03709	17.37824
		25 ng/ml BMP-7	2.011167	2.375610	.681	-4.15941	8.18174
Day_21	0 ng/ml BMP-7	25 ng/ml BMP-7	-15.911000*	4.237522	.005	-26.91784	-4.90416
		50 ng/ml BMP-7	-21.522000*	4.237522	<.001	-32.52884	-10.51516
	25 ng/ml BMP-7	0 ng/ml BMP-7	15.911000*	4.237522	.005	4.90416	26.91784
		50 ng/ml BMP-7	-5.611000	4.237522	.404	-16.61784	5.39584
	50 ng/ml BMP-7	0 ng/ml BMP-7	21.522000*	4.237522	<.001	10.51516	32.52884
		25 ng/ml BMP-7	5.611000	4.237522	.404	-5.39584	16.61784

*. The mean difference is significant at the 0.05 level.

(d)

Figure S2. Statistical analysis of WJMSC proliferation in DHDP scaffolds without BMP-7 (0ng/ml BMP-7) and with two BMP-7 concentrations (25ng/ml and 50ng/ml BMP-7) on day 7, 14 and 21: (a) normality test (Shapiro–Wilk), (b) homogeneity test (Levene), (c) one-way ANOVA, and (d) post hoc Tukey test. All p-values are presented. The data were normally distributed ($p > 0.05$) and homogeneous ($p > 0.05$). One-way ANOVA indicated a significant difference among groups ($p < 0.05$). Post hoc Tukey analysis showed that treatment with 25ng/mL and 50ng/mL BMP-7 resulted in significantly higher WJMSC proliferation compared with 0ng/mL BMP-7 ($p < 0.05$) on day 7, 14 and 21.

Tests of Normality

	Group	Kolmogorov-Smirnov[a] Statistic	df	Sig.	Shapiro-Wilk Statistic	df	Sig.
DMP1_Day21	0ng/ml BMP-7	.221	6	.200*	.921	6	.509
	25ng/ml BMP-7	.136	6	.200*	.979	6	.946
	50ng/ml BMP-7	.228	6	.200*	.877	6	.257
Runx2_Day21	0ng/ml BMP-7	.150	6	.200*	.957	6	.796
	25ng/ml BMP-7	.154	6	.200*	.987	6	.980
	50ng/ml BMP-7	.297	6	.107	.832	6	.112
DSPP_Day21	0ng/ml BMP-7	.265	6	.200*	.845	6	.144
	25ng/ml BMP-7	.238	6	.200*	.869	6	.222
	50ng/ml BMP-7	.197	6	.200*	.929	6	.574

*. This is a lower bound of the true significance.

a. Lilliefors Significance Correction

(a)

Tests of Homogeneity of Variances

		Levene Statistic	df1	df2	Sig.
DMP1_Day21	Based on Mean	2.606	2	15	.107
	Based on Median	1.583	2	15	.238
	Based on Median and with adjusted df	1.583	2	8.480	.261
	Based on trimmed mean	2.390	2	15	.126
Runx2_Day21	Based on Mean	.336	2	15	.720
	Based on Median	.324	2	15	.728
	Based on Median and with adjusted df	.324	2	11.432	.730
	Based on trimmed mean	.335	2	15	.720
DSPP_Day21	Based on Mean	45.181	2	15	<.001
	Based on Median	42.403	2	15	<.001
	Based on Median and with adjusted df	42.403	2	6.202	<.001
	Based on trimmed mean	44.799	2	15	<.001

(b)

ANOVA

		Sum of Squares	df	Mean Square	F	Sig.
DMP1_Day21	Between Groups	.000	2	.000	3.370	.062
	Within Groups	.001	15	.000		
	Total	.001	17			
Runx2_Day21	Between Groups	.006	2	.003	14.881	<.001
	Within Groups	.003	15	.000		
	Total	.009	17			
DSPP_Day21	Between Groups	.000	2	.000	7.761	.005
	Within Groups	.000	15	.000		
	Total	.001	17			

(c)

Multiple Comparisons

Games-Howell

Dependent Variable	(I) Group	(J) Group	Mean Difference (I-J)	Std. Error	Sig.	95% Confidence Interval Lower Bound	95% Confidence Interval Upper Bound
DMP1_Day21	0ng/ml BMP-7	25ng/ml BMP-7	-.00286617	.00395346	.756	-.0139797	.0082474
		50ng/ml BMP-7	.00565883	.00346186	.303	-.0049522	.0162699
	25ng/ml BMP-7	0ng/ml BMP-7	.00286617	.00395346	.756	-.0082474	.0139797
		50ng/ml BMP-7	.00852500*	.00242653	.022	.0014335	.0156165
	50ng/ml BMP-7	0ng/ml BMP-7	-.00565883	.00346186	.303	-.0162699	.0049522
		25ng/ml BMP-7	-.00852500*	.00242653	.022	-.0156165	-.0014335
Runx2_Day21	0ng/ml BMP-7	25ng/ml BMP-7	-.03985833*	.00866900	.003	-.0638287	-.0158879
		50ng/ml BMP-7	-.00327333	.00724751	.895	-.0231755	.0166288
	25ng/ml BMP-7	0ng/ml BMP-7	.03985833*	.00866900	.003	.0158879	.0638287
		50ng/ml BMP-7	.03658500*	.00835121	.004	.0132910	.0598790
	50ng/ml BMP-7	0ng/ml BMP-7	.00327333	.00724751	.895	-.0166288	.0231755
		25ng/ml BMP-7	-.03658500*	.00835121	.004	-.0598790	-.0132910
DSPP_Day21	0ng/ml BMP-7	25ng/ml BMP-7	-.01082283	.00400927	.090	-.0237097	.0020640
		50ng/ml BMP-7	.00067033	.00054153	.480	-.0010446	.0023852
	25ng/ml BMP-7	0ng/ml BMP-7	.01082283	.00400927	.090	-.0020640	.0237097
		50ng/ml BMP-7	.01149317	.00397547	.074	-.0014358	.0244222
	50ng/ml BMP-7	0ng/ml BMP-7	-.00067033	.00054153	.480	-.0023852	.0010446
		25ng/ml BMP-7	-.01149317	.00397547	.074	-.0244222	.0014358

*. The mean difference is significant at the 0.05 level.

(d)

Figure S3: Statistical analysis of relative dentinogenic gene expression (DMP-1, Runx2, and DSPP) in WJMSCs cultured on DHDP scaffolds without BMP-7 (0ng/mL) and with two BMP-7 concentrations (25ng/mL and 50ng/mL) on day 21. The analyses include (a) normality test (Shapiro–Wilk), (b) homogeneity test (Levene), (c) one-way ANOVA, and (d) post hoc Games–Howell test. All p-values are presented. The data were normally distributed ($p > 0.05$). Homogeneity of variance was met for DMP-1 and Runx2 ($p > 0.05$), but not for DSPP ($p < 0.05$). Although only DSPP violated the homogeneity assumption, the Games–Howell post hoc test was applied to all three genes to maintain a consistent and conservative analytical approach across the dataset. One-way ANOVA showed significant group differences for Runx2 and DSPP ($p < 0.05$). Post hoc Games–Howell analysis indicated that 25ng/mL BMP-7 resulted in significantly higher DMP-1 expression compared with 50ng/mL BMP-7 ($p = 0.022$). For Runx2, 25ng/mL BMP-7 showed significantly higher expression compared with 0 ng/mL ($p = 0.003$) and 50ng/mL BMP-7 ($p = 0.004$). No significant differences were found among groups for DSPP.

Tests of Normality

	Group	Kolmogorov-Smirnov[a] Statistic	df	Sig.	Shapiro-Wilk Statistic	df	Sig.
ELISA_DMP1_Day21	0ng/ml BMP-7	.293	6	.117	.819	6	.086
	25ng/ml BMP-7	.255	6	.200*	.902	6	.388
	50ng/ml MP-7	.168	6	.200*	.955	6	.777

*. This is a lower bound of the true significance.

a. Lilliefors Significance Correction

(a)

Tests of Homogeneity of Variances

		Levene Statistic	df1	df2	Sig.
ELISA_DMP1_Day21	Based on Mean	2.744	2	15	.096
	Based on Median	2.083	2	15	.159
	Based on Median and with adjusted df	2.083	2	9.960	.175
	Based on trimmed mean	2.622	2	15	.106

(b)

ANOVA

ELISA_DMP1_Day21

	Sum of Squares	df	Mean Square	F	Sig.
Between Groups	4.329	2	2.164	14.283	<.001
Within Groups	2.273	15	.152		
Total	6.602	17			

(c)

Post Hoc Tests

Multiple Comparisons

Dependent Variable: ELISA_DMP1_Day21

Tukey HSD

(I) Group	(J) Group	Mean Difference (I-J)	Std. Error	Sig.	95% Confidence Interval Lower Bound	Upper Bound
0ng/ml BMP-7	25ng/ml BMP-7	-1.15733333*	.22475329	<.001	-1.7411233	-.5735434
	50ng/ml MP-7	-.30000000	.22475329	.399	-.8837900	.2837900
25ng/ml BMP-7	0ng/ml BMP-7	1.15733333*	.22475329	<.001	.5735434	1.7411233
	50ng/ml MP-7	.85733333*	.22475329	.005	.2735434	1.4411233
50ng/ml MP-7	0ng/ml BMP-7	.30000000	.22475329	.399	-.2837900	.8837900
	25ng/ml BMP-7	-.85733333*	.22475329	.005	-1.4411233	-.2735434

*. The mean difference is significant at the 0.05 level.

(d)

Figure S4. Statistical analysis of DMP-1 protein expression between without BMP-7 treatment (0ng/ml) and two concentrations of BMP-7 treatment (25ng/ml and 50ng/ml) on day 21: (a) normality test (Shapiro–Wilk), (b) homogeneity test (Levene), (c) one-way ANOVA, and (d) post hoc Tukey test. All p-values are presented. The data were normally distributed ($p > 0.05$) and homogeneous ($p > 0.05$). One-way ANOVA indicated a significant difference among groups ($p < 0.05$). Post hoc Tukey analysis showed that treatment with 25ng/mL BMP-7 resulted in significantly higher DMP-1 protein expression compared with 0ng/mL BMP-7 ($p < 0.001$) and 50ng/mL BMP-7 ($p = 0.005$).

Tests of Normality

	Group	Kolmogorov-Smirnov[a] Statistic	df	Sig.	Shapiro-Wilk Statistic	df	Sig.
ELISA_DSPP_Day21	0ng/ml BMP-7	.178	6	.200*	.976	6	.928
	25ng/ml BMP-7	.227	6	.200*	.944	6	.693
	50ng/ml BMP-7	.203	6	.200*	.904	6	.395

*. This is a lower bound of the true significance.

a. Lilliefors Significance Correction

(a)

Tests of Homogeneity of Variances

		Levene Statistic	df1	df2	Sig.
ELISA_DSPP_Day21	Based on Mean	12.871	2	15	<.001
	Based on Median	11.169	2	15	.001
	Based on Median and with adjusted df	11.169	2	9.297	.003
	Based on trimmed mean	12.706	2	15	<.001

(b)

ANOVA

ELISA_DSPP_Day21

	Sum of Squares	df	Mean Square	F	Sig.
Between Groups	58141.626	2	29070.813	139.055	<.001
Within Groups	3135.900	15	209.060		
Total	61277.526	17			

(c)

Post Hoc Tests

Multiple Comparisons

Dependent Variable: ELISA_DSPP_Day21

Games-Howell

(I) Group	(J) Group	Mean Difference (I-J)	Std. Error	Sig.	95% Confidence Interval Lower Bound	Upper Bound
0ng/ml BMP-7	25ng/ml BMP-7	-125.229833*	9.633548	<.001	-155.87998	-94.57969
	50ng/ml BMP-7	-9.950667	3.841623	.078	-21.10851	1.20717
25ng/ml BMP-7	0ng/ml BMP-7	125.229833*	9.633548	<.001	94.57969	155.87998
	50ng/ml BMP-7	115.279167*	10.074556	<.001	84.76879	145.78954
50ng/ml BMP-7	0ng/ml BMP-7	9.950667	3.841623	.078	-1.20717	21.10851
	25ng/ml BMP-7	-115.279167*	10.074556	<.001	-145.78954	-84.76879

*. The mean difference is significant at the 0.05 level.

(d)

Figure S5. Statistical analysis of DSPP protein expression in WJMSCs cultured without BMP-7 (0 ng/mL) and with two BMP-7 concentrations (25 ng/mL and 50 ng/mL) on day 21. The analyses include (a) normality test (Shapiro–Wilk), (b) homogeneity test (Levene), (c) one-way ANOVA, and (d) post hoc Games–Howell test. All p-values are reported. The data were normally distributed ($p > 0.05$) but did not meet the homogeneity assumption ($p < 0.05$). One-way ANOVA indicated a significant difference among groups ($p < 0.05$). Post hoc Games–Howell analysis showed that 25 ng/mL BMP-7 treatment resulted in significantly higher DSPP protein expression compared with both 0 ng/mL BMP-7 ($p < 0.001$) and 50 ng/mL BMP-7 ($p < 0.001$).
